# Supplementary material for: The clonal structure and dynamics of the human T cell response to an organic chemical hapten
Source: eLife. 2021 Jan 12;10:e54747. doi: 10.7554/eLife.54747 (PMC7880692; doi:10.7554/eLife.54747)
Supplement: Supplementary file 1. — ‘_TRA’ are the TCR alpha chain samples, and ‘_TRB’ the beta chain samples. [file elife-54747-supp1.docx]

Supplementary File 1

| Sample | Unique TCRs | Total TCRs |
| --- | --- | --- |
| 003_PS_TRA | 271,042 | 628,446 |
| 003_PS_TRB | 328,242 | 690,600 |
| 003_PT1_TRA | 269,066 | 644,992 |
| 003_PT1_TRB | 319,697 | 677,282 |
| 003_PT2_TRA | 268,911 | 586,024 |
| 003_PT2_TRB | 335,290 | 650,535 |
| 004_PS_TRA | 261,113 | 372,310 |
| 004_PS_TRB | 342,336 | 452,889 |
| 004_PT1_TRA | 255,419 | 361,495 |
| 004_PT1_TRB | 333,472 | 433,910 |
| 004_PT2_TRA | 55,389 | 65,284 |
| 004_PT2_TRB | 47,949 | 54,794 |
| 004_PT3_TRA | 78,803 | 94,056 |
| 004_PT3_TRB | 87,886 | 102,424 |
| 005_PS_TRA | 28492 | 42155 |
| 005_PS_TRB | 41273 | 70559 |
| 005_PT1_TRA | 28,317 | 44,541 |
| 005_PT1_TRB | 26,257 | 45,179 |
| 005_PT3_TRA | 32601 | 47921 |
| 005_PT3_TRB | 47004 | 81470 |
| 006_PS_TRA | 53741 | 90750 |
| 006_PS_TRB | 70376 | 124769 |
| 006_PT1_TRA | 48,699 | 77,925 |
| 006_PT1_TRB | 58,116 | 94,298 |
| 006_PT2_TRA | 35,086 | 51,744 |
| 006_PT2_TRB | 25,839 | 37,594 |
| 006_PT3_TRA | 60,932 | 97,987 |
| 006_PT3_TRB | 75,730 | 121,982 |
| 007_PS_TRA | 59,593 | 80,175 |
| 007_PS_TRB | 57,493 | 76,126 |
| 007_PT1_TRA | 64,375 | 86,328 |
| 007_PT1_TRB | 64,030 | 82,703 |
| 007_PT2_TRA | 61,059 | 81,059 |
| 007_PT2_TRB | 64,443 | 83,226 |
| 007_PT3_TRA | 101,207 | 155,417 |
| 007_PT3_TRB | 77,278 | 101,151 |
| 008_PS_TRA | 36,514 | 45,559 |
| 008_PS_TRB | 37,186 | 46,047 |
| 008_PT1_TRA | 50,819 | 62,951 |
| 008_PT1_TRB | 48,215 | 57,082 |
| 008_PT2_TRA | 53,367 | 64,964 |
| 008_PT2_TRB | 51,106 | 60,405 |
| 010_PT3_TRA | 107,788 | 136,556 |
| 010_PT3_TRB | 169,860 | 213,511 |
| 011_PT3_TRA | 56,539 | 68,190 |
| 011_PT3_TRB | 66,702 | 79,272 |
| 013_PT3_TRA | 107,685 | 134,176 |
| 013_PT3_TRB | 103,860 | 125,657 |
| 014_PS_TRA | 39,601 | 59,351 |
| 014_PS_TRB | 34,253 | 47,031 |
| 014_PT1_TRA | 33,384 | 56,132 |
| 014_PT1_TRB | 35,906 | 54,433 |
| 014_PT2_TRA | 36001 | 52662 |
| 014_PT2_TRB | 49134 | 72362 |
| 015_PS_TRA | 213,202 | 323,160 |
| 015_PS_TRB | 322,678 | 466,885 |
| 015_PT1_TRA | 96,488 | 123,470 |
| 015_PT1_TRB | 122,768 | 153,478 |
| 015_PT2_TRA | 83,733 | 109,941 |
| 015_PT2_TRB | 111,651 | 143,484 |
| 015_PT3_TRA | 113,418 | 146,627 |
| 015_PT3_TRB | 214,135 | 284,117 |
| 016_PS_TRA | 68,605 | 95,332 |
| 016_PS_TRB | 57,725 | 88,027 |
| 016_PT1_TRA | 56,588 | 84,855 |
| 016_PT1_TRB | 65,236 | 97,909 |
| 016_PT2_TRA | 45,675 | 69,049 |
| 016_PT2_TRB | 48,117 | 72,379 |
| 016_PT3_TRA | 55,636 | 87,581 |
| 016_PT3_TRB | 64,151 | 101,009 |
| 017_PS_TRA | 90,604 | 144,808 |
| 017_PS_TRB | 104,766 | 158,350 |
| 017_PT1_TRA | 52,132 | 76,154 |
| 017_PT1_TRB | 55,794 | 78,679 |
| 017_PT2_TRA | 97,916 | 149,620 |
| 017_PT2_TRB | 98,023 | 151,015 |
| 018_PS_TRA | 128,182 | 165,690 |
| 018_PS_TRB | 145,553 | 179,601 |
| 019_PS_TRA | 82,912 | 116,951 |
| 019_PS_TRB | 77,720 | 112,083 |
| 019_PT1_TRA | 83,647 | 124,414 |
| 019_PT1_TRB | 93,404 | 135,862 |
| 020_PS_TRA | 123,176 | 178,327 |
| 020_PS_TRB | 133,753 | 188,523 |
| 020_PT1_TRA | 160,178 | 233,985 |
| 020_PT1_TRB | 197,797 | 279,109 |
| 020_PT2_TRA | 65,294 | 91,476 |
| 020_PT2_TRB | 79,020 | 109,570 |
| 020_PT3_TRA | 81,636 | 105,771 |
| 020_PT3_TRB | 94,635 | 119,064 |
| 021_PS_TRA | 214,727 | 276,925 |
| 021_PS_TRB | 327,709 | 404,403 |
| 021_PT1_TRA | 133,108 | 160,729 |
| 021_PT1_TRB | 158,467 | 182,688 |
| 021_PT2_TRA | 84,889 | 98,908 |
| 021_PT2_TRB | 101,614 | 114,593 |
| 022_PS_TRA | 126,089 | 154,813 |
| 022_PS_TRB | 193,310 | 238,596 |
| 022_PT1_TRA | 183,022 | 225,569 |
| 022_PT1_TRB | 197,407 | 232,453 |
| 022_PT2_TRA | 92,111 | 106,028 |
| 022_PT2_TRB | 95,404 | 107,643 |
| 022_PT3_TRA | 67,790 | 76,451 |
| 022_PT3_TRB | 78,763 | 87,178 |
| 023_PS_TRA | 107,739 | 127,237 |
| 023_PS_TRB | 128,500 | 147,531 |
| 023_PT1_TRA | 119,245 | 141,309 |
| 023_PT1_TRB | 133,611 | 154,344 |
| 023_PT2_TRA | 61,838 | 69,446 |
| 023_PT2_TRB | 68,725 | 75,778 |
| 023_PT3_TRA | 69,671 | 77,262 |
| 023_PT3_TRB | 83,357 | 91,107 |
| 024_PS_TRA | 124,983 | 186,837 |
| 024_PS_TRB | 127,746 | 182,537 |
| 024_PT1_TRA | 91,411 | 126,165 |
| 024_PT1_TRB | 89,129 | 122,759 |
| 024_PT2_TRA | 47,239 | 63,876 |
| 024_PT2_TRB | 50,330 | 66,015 |
| 024_PT3_TRA | 58,581 | 78,931 |
| 024_PT3_TRB | 63,012 | 81,826 |
| 025_PS_TRA | 32014 | 38125 |
| 025_PS_TRB | 42470 | 49864 |
| 025_PT1_TRA | 57,118 | 68,077 |
| 025_PT1_TRB | 63,470 | 74,129 |
| 025_PT3_TRA | 28,438 | 32,834 |
| 025_PT3_TRB | 49,611 | 58,096 |
| 026_PS_TRA | 73702 | 86697 |
| 026_PS_TRB | 103193 | 120025 |
| 026_PT1_TRA | 74,491 | 89,861 |
| 026_PT1_TRB | 93,665 | 110,365 |
| 027_PT1_TRA | 19,667 | 24,445 |
| 027_PT1_TRB | 19,095 | 23,224 |
| 028_PS_TRA | 41,555 | 54,199 |
| 028_PS_TRB | 49,017 | 61,219 |
| 028_PT2_TRA | 29,455 | 40,423 |
| 028_PT2_TRB | 33,369 | 45,546 |
| 028_PT3_TRA | 36874 | 56281 |
| 028_PT3_TRB | 44114 | 66172 |
| 029_PS_TRA | 37,976 | 42,222 |
| 029_PS_TRB | 46,915 | 51,974 |
| 029_PT1_TRA | 82,180 | 95,943 |
| 029_PT1_TRB | 98,752 | 113,345 |
| 029_PT2_TRA | 114,289 | 141,074 |
| 029_PT2_TRB | 148,514 | 178,007 |
| 029_PT3_TRA | 71,513 | 88,642 |
| 029_PT3_TRB | 89,723 | 108,502 |
| 030_PS_TRA | 44,344 | 53,591 |
| 030_PS_TRB | 65,928 | 82,510 |
| 030_PT1_TRA | 45,745 | 54,961 |
| 030_PT1_TRB | 50,838 | 61,558 |
| 030_PT3_TRA | 78,566 | 100,427 |
| 030_PT3_TRB | 97,172 | 124,366 |
| 031_PS_TRA | 41,853 | 54,634 |
| 031_PS_TRB | 51,488 | 68,567 |
| 033_PS_TRA | 76,829 | 97,615 |
| 033_PS_TRB | 93,931 | 116,781 |
| 033_PT1_TRA | 67,073 | 85,465 |
| 033_PT1_TRB | 76,613 | 93,577 |
| 033_PT2_TRA | 95,536 | 120,863 |
| 033_PT2_TRB | 121,011 | 148,432 |
| 034_PS_TRA | 46,002 | 58,417 |
| 034_PS_TRB | 62,906 | 77,814 |
| 034_PT1_TRA | 82,928 | 114,661 |
| 034_PT1_TRB | 119,963 | 158,542 |
| 034_PT2_TRA | 78,668 | 100,281 |
| 034_PT2_TRB | 101,602 | 125,581 |
| HV1_PS_TRA | 53766 | 64027 |
| HV1_PS_TRB | 54715 | 62205 |
| HV1_PT1_TRA | 112324 | 135447 |
| HV1_PT1_TRB | 93427 | 109359 |
| HV1_PT2_TRA | 51340 | 61286 |
| HV1_PT2_TRB | 53579 | 60629 |
| HV2_PS_TRA | 60100 | 101095 |
| HV2_PS_TRB | 57003 | 88529 |
| HV2_PT1_TRA | 21667 | 33340 |
| HV2_PT1_TRB | 19673 | 28413 |
| HV2_PT2_TRA | 24479 | 38833 |
| HV2_PT2_TRB | 21151 | 32403 |
| HV3_PS_TRA | 56640 | 71050 |
| HV3_PS_TRB | 62540 | 74893 |
| HV3_PT1_TRA | 87495 | 110940 |
| HV3_PT1_TRB | 65333 | 78492 |
| HV3_PT2_TRA | 54700 | 69410 |
| HV3_PT2_TRB | 44113 | 52951 |
| HV4_PS_TRA | 76346 | 94873 |
| HV4_PS_TRB | 80472 | 94672 |
| HV4_PT1_TRA | 30694 | 36756 |
| HV4_PT1_TRB | 28111 | 33324 |
| HV4_PT2_TRA | 25577 | 32495 |
| HV4_PT2_TRB | 28909 | 35651 |
| HV5_PS_TRA | 83525 | 119551 |
| HV5_PS_TRB | 96043 | 127397 |
| HV5_PT1_TRA | 28018 | 35306 |
| HV5_PT1_TRB | 26015 | 31189 |
| HV5_PT2_TRB | 53971 | 66672 |

**Supplementary Table 1**: **Unique and total TCR numbers for each TCRseq sample.** ‘_TRA’ are the TCR alpha chain samples, and ‘_TRB’ the beta chain samples.
