# Supplementary material for: The clonal structure and dynamics of the human T cell response to an organic chemical hapten
Source: eLife. 2021 Jan 12;10:e54747. doi: 10.7554/eLife.54747 (PMC7880692; doi:10.7554/eLife.54747)
Supplement: Supplementary file 2. — (A) The Shannon diversity index of the healthy volunteers (n = 14 samples from five individuals), pre-sensitization (n = 25), and post-sensitization (n = 58; from all three time points) TCR repertoire samples. All samples were randomly subsampled to the minimum sample size (20,172 alpha TCRs), and the Shannon diversity index of the subsample was then calculated. Each sample is represented by a dot. The box plots show the median, and lower and upper quartiles of each group. Differences in the distribution of the three groups were tested using a Kruskal–Wallis rank sum test and were non-significant (p=0.7658). (B) The Gini inequality coefficient of the healthy volunteers, pre-sensitization and post-sensitization alpha chain TCR repertoire samples, subsampled as in (A). Differences in the distribution of the three groups were tested using a Kruskal–Wallis rank sum test and were non-significant (p=0.9062). (C) The number of alpha TCRs that appear with a frequency of 1/1000 or higher in each sample (termed ‘abundant TCRs’), for the healthy volunteers, pre-sensitization and post-sensitization samples, subsampled as in (A) and (B). A Kruskal–Wallis rank sum test revealed no statistical difference between the groups (p=0.9256). (D) – (F) The alpha chains of the sensitized samples were separated according to time point: PT1 (n = 23), PT2 (n = 18), and PT3 (n = 17). The Shannon diversity index (D), the Gini coefficient (E), and the number of abundant clones (F) of these subsamples were calculated. Kruskal–Wallis rank sum tests were used to compare between the three groups in each case. All tests showed no statistically significant difference, with p=0.9608, p=0.9281, and p=0.8681 respectively. [file elife-54747-supp2.docx]

Supplementary File 2

Supplementary Figure 1: Repeated exposure to DPC does not alter the global structure of the peripheral blood TCR repertoire. (A) The Shannon diversity index of the healthy volunteers (n=14 samples from five individuals), pre-sensitization (n=25) and post-sensitization (n=58; from all three time points) TCR repertoire samples. All samples were randomly subsampled to the minimum sample size (20,172 alpha TCRs), and the Shannon diversity index of the subsample was then calculated. Each sample is represented by a dot. The box plots show the median, and lower and upper quartiles of each group. Differences in the distribution of the three groups were tested using a Kruskal-Wallis rank sum test and were non-significant (p=0.7658). (B) The Gini inequality coefficient of the healthy volunteers, pre-sensitization and post-sensitization alpha chain TCR repertoire samples, subsampled as in (A). Differences in the distribution of the three groups were tested using a Kruskal-Wallis rank sum test and were non-significant (p=0.9062). (C) The number of alpha TCRs that appear with a frequency of 1/1000 or higher in each sample (termed ‘abundant TCRs’), for the healthy volunteers, pre-sensitization and post-sensitization samples, subsampled as in (A) and (B). A Kruskal-Wallis rank sum test revealed no statistical difference between the groups (p=0.9256). (D)-(F) The alpha chains of the sensitized samples were separated according to time point: PT1 (n=23), PT2 (n=18) and PT3 (n=17). The Shannon diversity index (D), the Gini coefficient (E) and the number of abundant clones (F) of these subsamples were calculated. Kruskal-Wallis rank sum tests were used to compare between the three groups in each case. All tests showed no statistically significant difference, with p=0.9608, p=0.9281 and p=0.8681 respectively.
