# Supplementary material for: The clonal structure and dynamics of the human T cell response to an organic chemical hapten
Source: eLife. 2021 Jan 12;10:e54747. doi: 10.7554/eLife.54747 (PMC7880692; doi:10.7554/eLife.54747)
Supplement: Supplementary file 3. — (A) The abundance distribution of TCRs at PS and PT1. All samples were subsampled to the same number of TCRs (28,000). Each unique TCR is represented by a dot, and the axes represent the number of times it is observed in the PS (x-axis) and PT1 (y-axis) sample of the same individual, and equally spaced time points for the healthy volunteers. The pink dots identify a population of TCRs absent in the PS sample and expanded (abundance ≥8) in the PT1 sample. The blue dots identify a population of TCRs absent in the PT1 sample and expanded (≥8) in the PS sample. The numbers indicate the percentage of PT1 expanded TCRs (pink) and PS expanded TCRs (blue). (B) The correlation between the percentage of PT1 expanded alpha chain TCRs (x-axis) and the percentage of PT1 expanded beta chain TCRs (y-axis) for each individual (n = 22), subsampled as in (A). Spearman’s rho = 0.85, p<0.0001. The line x=y is shown. [file elife-54747-supp3.docx]

Supplementary File 3

Supplementary Figure 2: Sensitization with DPC induces a transient expansion in the frequency of a small subset of the TCR repertoire. (A) The abundance distribution of TCRs at PS and PT1. All samples were subsampled to the same number of TCRs (28,000). Each unique TCR is represented by a dot, and the axes represent the number of times it is observed in the PS (x-axis) and PT1 (y-axis) sample of the same individual, and equally spaced time points for the healthy volunteers. The pink dots identify a population of TCRs absent in the PS sample and expanded (abundance ≥ 8) in the PT1 sample. The blue dots identify a population of TCRs absent in the PT1 sample and expanded (≥ 8) in the PS sample. The numbers indicate the percentage of PT1 expanded TCRs (pink) and PS expanded TCRs (blue). (B) The correlation between the percentage of PT1 expanded alpha chain TCRs (x-axis) and the percentage of PT1 expanded beta chain TCRs (y-axis) for each individual (n=22), subsampled as in (A). Spearman’s rho=0.85, p<0.0001. The line x=y is shown.
