# Supplementary material for: The clonal structure and dynamics of the human T cell response to an organic chemical hapten
Source: eLife. 2021 Jan 12;10:e54747. doi: 10.7554/eLife.54747 (PMC7880692; doi:10.7554/eLife.54747)
Supplement: Supplementary file 4. — (A) The abundances of the PT1 expanded (threshold ≥8) alpha TCRs at the four time points: PS, PT1, PT2, and PT3. Each panel is a different patient (n = 10). (B) The abundances of the PT2 expanded (threshold ≥8) alpha TCRs at the four time points: PS, PT1, PT2, and PT3. Each panel is a different patient (n = 10). (C) Equivalent time points (0 weeks, 2 weeks, and 6 weeks) for four healthy volunteers for whom we had all three times points. Top row is PT1 expanded alpha TCRs; bottom row is PT2 expanded alpha TCRs. The fourth volunteer had no PT1 expanded alpha TCRs. The fifth volunteer had no PT1 expanded alpha TCRs and no PT2 alpha sample. [file elife-54747-supp4.docx]

***Supplementary File 4***

Supplementary Figure 3: Dynamic changes in TCR frequency following sensitization. (A) The abundances of the PT1 expanded (threshold ≥ 8) alpha TCRs at the four time points: PS, PT1, PT2, and PT3. Each panel is a different patient (n=10). (B) The abundances of the PT2 expanded (threshold ≥ 8) alpha TCRs at the four time points: PS, PT1, PT2, and PT3. Each panel is a different patient (n=10). (C) Equivalent time points (0 weeks, 2 weeks and 6 weeks) for four healthy volunteers for whom we had all three times points. Top row is PT1 expanded alpha TCRs; bottom row is PT2 expanded alpha TCRs. The fourth volunteer had no PT1 expanded alpha TCRs. The fifth volunteer had no PT1 expanded alpha TCRs and no PT2 alpha sample.
