# Supplementary material for: The clonal structure and dynamics of the human T cell response to an organic chemical hapten
Source: eLife. 2021 Jan 12;10:e54747. doi: 10.7554/eLife.54747 (PMC7880692; doi:10.7554/eLife.54747)
Supplement: Supplementary file 5. — The CDR3s of the largest cluster of PT1 expanded CDR3 beta sequences (see Figure 6D and E) were aligned using the MUSCLE alignment algorithm in Aliview (https://ormbunkar.se/aliview/). [file elife-54747-supp5.docx]

Supplementary File 5


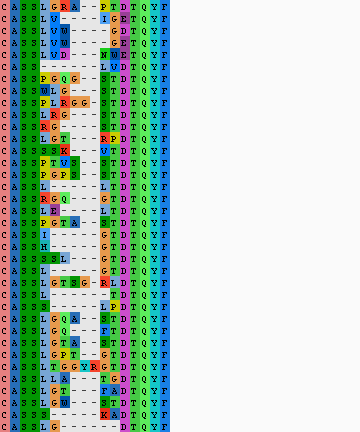


Supplementary Figure 4: Sequence alignment of the CDR3 sequences from the largest cluster of TCR beta PT1 expanded CDR3s. The CDR3s of the largest cluster of PT1 expanded CDR3 beta sequences (see Figure 6D and E) were aligned using the MUSCLE alignment algorithm in Aliview (https://ormbunkar.se/aliview/).
